# A high-throughput drug discovery pipeline to optimize kidney normothermic machine perfusion

**DOI:** 10.3389/fphys.2022.974615

**Published:** 2022-09-26

**Authors:** Smilla Hofmann, Florian Grahammer, Ilka Edenhofer, Victor G. Puelles, Tobias B. Huber, Jan Czogalla

**Affiliations:** ^1^ III. Department of Medicine, University Medical Center Hamburg-Eppendorf UKE, Hamburg, Germany; ^2^ University Transplant Center, University Medical Center Hamburg-Eppendorf UKE, Hamburg, Germany; ^3^ Department of Clinical Medicine, Division of Pathology, Aarhus University, Aarhus, Denmark

**Keywords:** normothermic machine perfusion, kidney transplantation, kidney regeneration, er stress, NADH, upr, AKI

## Abstract

Kidney transplantation is the only definitive therapy for end-stage kidney disease. The shortage of organs for transplantation is the main limitation of this life-saving treatment. Normothermic machine perfusion (NMP) is a novel preservation technique with the potential to increase the number of transplantable kidneys through reducing delayed graft function and organ evaluation under physiological conditions. To date, the cellular effects and possible pharmacological interventions during machine perfusion are incompletely understood. A major limitation is the technically complex, time-consuming, and small-scale replication of NMP in rodent models. To overcome this, we developed a 3D-printed, high throughput *ex-vivo* mouse kidney slice incubator (KSI) mimicking mouse kidney NMP by working under closely resembling conditions. KSI significantly reduced the time per experiment and increased the sample throughput (theoretical: 54 incubations with *n* = 500/day). The model recapitulated the cellular responses during NMP, namely increased endoplasmic reticulum stress (ER stress). Using KSI, five pharmacological interventions against ER stress taken from the literature were tested. While four were ineffective and excluded, one, β-Nicotinamide-adenine-dinucleotide (NADH), ameliorated ER stress significantly during KSI. The test of NADH in mouse kidney NMP replicated the positive effects against ER stress. This suggests that testing the addition of NADH during clinical kidney NMP might be warranted.

## 1 Introduction

End-stage kidney disease (ESKD) is a global health burden with four to seven million estimated patients in need of renal replacement therapy worldwide. The increasing prevalence of risk factors for ESKD, such as hypertension, diabetes mellitus, obesity, and general aging of the population, further drives the incidence of ESKD (Lv and Zhang, 2019). Kidney transplantation is the gold standard for the treatment of ESKD, as the mortality of patients who receive a renal transplant is lower and the quality of life is greater compared to dialysis patients (Tonelli et al., 2011; Wyld et al., 2012). A growing organ shortage, resulting from fewer donations and increased incidence of ESKD, presents a major problem in the field of kidney transplantation (Liyanage et al., 2015). One solution may be increased use of marginal organs, organs in less than an optimal condition that would normally not be transplanted (Noble et al., 2019). However, these organs are more prone to ischemic injury, delayed graft function, and associated long-term complications (Giraud et al., 2020).

Kidney normothermic machine perfusion (NMP) has gained attention as a promising preservation technique during transplantation due to the theoretical advantages it offers in improving ischemic damage, as well as organ conditioning, analysis, and treatment (Hosgood et al., 2021). NMP for human kidneys has only been tested in-depth in a small number of studies. However, the molecular readout analyzed in these studies unequivocally suggested cytokine release and metabolic- and ER stress during perfusion, revealing the importance of further improvement of the buffer before routine clinical application (Hameed et al., 2019; Ferdinand et al., 2021).

We have recently established a cell-free rodent NMP model for the rigorous preclinical study of potential interventions during NMP (Czogalla et al., 2021). However, the complexity and time-consuming nature of rodent NMP models only allows small-scale sample generation, resulting in slow progress. Here, we describe the generation of a novel, high-throughput *ex-vivo* mouse kidney slice incubation model (KSI) working under “NMP-like” conditions, which recapitulates ER stress as seen during NMP and can be used for drug pre-screening before the time-consuming testing in NMP.

## 2 Methods

### 2.1 Animals

Animal experiments were carried out with the approval of the Hamburg University Hospital-Eppendorf and the Hamburg State Department for Animal Welfare (Ethics Approval No. N002/2020). Age- and weight-matched male wildtype C57/Bl6 mice were housed under a 12-h light-dark pattern in an accredited animal facility at UKE Hamburg with free access to chow and water.

### 2.2 Mouse kidney slice incubator

KSI consists of a beaker with an inset 3D printed chamber, with slots for up to nine kidney slices. Please see Supplementary Figure S2 for dimensions and attached. stl files for 3D-printables. For 3D printing, Extrudr GreenTec Pro natur was used (Extrudr, Austria) based on the basis of excellent oil and salt resistance. Gey’s Balanced Salt Solution (GBSS, G9779-6X500ML, Sigma-Aldrich) was used as incubation buffer. Silicon tubing connected to a standard aquarium bubble air stone was used to enrich the buffer with carbogen, a mixture of 95% O2 and 5% CO2, until saturation was reached. The beaker including the 3D-printed chamber was placed in a water bath heated to reach a constant temperature of 33°C. Decapsulated mouse kidneys were cut into 1-mm-sized slices with a homemade razorblade-based tissue chopper and got either incubated in drug solutions (GBSS + added drugs) or in control solution (GBSS + diluent, where used) for 30 min. After incubation, tissue was snap-frozen in liquid nitrogen, placed in 4% PFA for diffusion fixation, or RNAlater (AM7021, Qiagen) for mRNA analysis. Experiments were repeated in at least three animals. No further repeats were performed to satisfy animal ethics concerns.

### 2.3 Mouse kidney normothermic machine perfusion

Mouse kidney NMP was performed as previously described (Czogalla et al., 2016). After median laparotomy, ligatures were placed around aorta, kidney arteries and vena cava. The kidney artery and vein were cannulated and the kidney removed from the animal. The kidney was connected to a pressure-controlled perfusion circuit and perfusion was carried out for 1 h at 100 mmHg. The kidney was placed in a moist chamber (TYPE 834/8, 73-2901, Hugo Sachs, Germany) and the lid closed. Perfusion flow was continuously recorded at the arterial tip using a pressure transducer (APT300, Hugo Sachs, Germany), a three-channel cannula (73-3309, Hugo Sachs, Germany), a TAM-D Plugsys transducer (73-1793, Hugo Sachs, Germany) and recording software (HSE BDAS 73-4796, Hugo Sachs, Germany) (Supplementary Figure S3). The pump used was a Reglo Digital 4-Channel pump (75-1004, Hugo Sachs, Germany). Buffer reservoirs used were jacketed and filled with 37°C (73-3440). For further surgical and technical details, please refer to: (Czogalla et al., 2016). GBSS (Sigma-Aldrich, G9779) was used as perfusate, kept at 37°C and continuously enriched via dialysis with 95% O_2_ and 5% CO_2_. For the intervention, b-nicotinamide adenine dinucleotide (Sigma-Aldrich, N8129) was dissolved in GBSS to a concentration of 0.07 mM. Perfusion flow was continuously recorded at the arterial tip using a pressure transducer (APT300, Hugo Sachs, Germany), a three-channel cannula (73-3309, Hugo Sachs, Germany), a TAM-D plugsys transducer (73-1793, Hugo Sachs, Germany) and recording software (HSE BDAS 73-4796, Hugo Sachs, Germany) (Supplementary Figure S3). After perfusion, tissue was either snap-frozen in liquid nitrogen, placed in 4% PFA for diffusion-fixation, or RNAlater (AM7021, Qiagen) for mRNA analysis. Experiments were repeated in six animals.

**Table:** 

List of incubation drugs			
**Drug**	**Company**	**Article number**	**Concentration**
Salubrinal	Sigma Aldrich	SML0951	37.42 µM
Isoproterenol	Sigma Aldrich	I5627	200 nM
Ursodeoxycholic Acid	Sigma Aldrich	U5127	100 µM
β-Nicotinamide-adenine-dinucleotide	Sigma Aldrich	N8129	0.07 mM
Roflumilast	Sigma Aldrich	SML1099	49.6 µM
**List of components for KSI**			
**Components**	**Company**	**Article number**	
Glass beaker	Th. Geyer	7690006	
3D printed chamber	Self-made	Design in Supplement	
Gey’s Balanced Salt Solution	Sigma Aldrich	G9779-6X500 ML	
Printing material (GREENTEC PRO natur)	Extrudr	2286	
Bubble air stone	Aipaide	APD-Stone-063	
Water bath	Julabo	CD-BT19	

**Table:** 

List of antibodies			
**Target**	**Company**	**Article number**	**Dilution**
BiP	Cell Signaling	3183	1:500
pS51-eIF2α	Cell Signaling	9721	1:250
total eIF2α	Cell Signaling	2103S	1:500
alpha-tubulin	Sigma-Aldrich	T9026	1:5.000
beta-actin	Sigma-Aldrich	A5441	1:10.000

**Table:** 

List of primers			
**Target**	**Company**	**Article number**	**TaqMan Assay ID**
Mouse GAPD (GAPDH)	Thermo Fisher Scientific	4352932E	Mm99999915_g1
Mouse activating transcription factor 4 (ATF4)	Thermo Fisher Scientific	4331182	Mm00515324_m1
Mouse DNA damage inducible transcript 3 (CHOP)	Thermo Fisher Scientific	4331182	Mm00492097_m1
Mouse receptor-interacting serine-threonine kinase 3 (RIPK3)	Thermo Fisher Scientific	4331182	Mm00444947_m1

### 2.4 Immunoblotting

For Western blot analysis, kidney samples were lysed for 30 min in 0.5–1 ml of RIPA buffer with the Minilys personal homogenizer (P000673-MLYS0-A, Bertin Technologies). Subsequently, samples were centrifuged at 4.600 rpm for 15 min, the supernatant was transferred to a new tube for the adjustment of protein concentration. Protein content was determined by a BCA assay (23227, Thermo Fisher Scientific) with a Tecan sunrise scanner (30111999, Tecan Trading AG). Before protein electrophoresis, the samples were diluted with 2x Laemmli buffer (1:1) and cooked at 95°C for 5 min. For each lane, 50 µg of protein were loaded. The sample proteins and a protein ladder in one lane were separated in pre-cast gels of 4%–20% (4561096EDU, BioRad) for 20 min at 80 V and 1 h at 120 V. Immunoblotting was performed with Trans-Blot Turbo Mini 0.2 µm PVDF transfer packs (704156, BioRad) for 7 min at 25 V, 1.3 A, using a Trans-Blot Turbo Transfer System (1704150, BioRad). Blocking was performed with a 5% bovine-serum-albumin- (BSA, A9430, Sigma-Aldrich) PBST solution (0.1% Tween) for 1 h. The membranes were incubated with a primary antibody solution at 4°C overnight. Incubation with horseradish peroxidase-conjugated secondary antibodies was performed at room temperature for 1 h. Imaging was performed after incubation of the membranes with ECL Western Blot Substrate (32209, Thermo Scientific) with a chemiluminescence detector (Amersham Imager 600). Analysis was performed with ImageJ [2.3.0/1.53 f (64-bit), US National Institutes of Health].

### 2.5 Quantitative real-time RT-PCR

Total RNA was extracted with QIAzol Lysis Reagent (79306, Qiagen) and the isolation was performed with the miRNeasy Plus Universal Mini Kit (217084, Qiagen). The RNase-Free DNase Set (79254, Qiagen) was used for DNA digestion. For reverse transcription, Protoscript II First Strand cDNA Synthesis Kit (E6560L, New England BioLabs) was applied. Quantitative PCR was performed on a QuantStudio 3 System (A28566, Thermo Fisher Scientific) using 0.5 µg of cDNA, gene-specific TaqMan™ primers, and TaqMan™ Fast Universal PCR Master Mix (43-660-73, Thermo Fisher Scientific). Glyceraldehyde-3-phosphate dehydrogenase (GAPDH, Thermo Fisher Scientific) was used for normalization. The qPCR data were analyzed with the double delta CT method.

### 2.6 Histological analysis

Samples diffusion-fixed in 4% formaldehyde and stored at 4°C were embedded in paraffin and stained with periodic acid-Schiff (PAS) reagent following standard protocol. Incubation times were 1 hour in Schiff’s reagent and staining of nuclei in hematoxylin for 3 min. Tubular damage was evaluated by two researchers. For TUNEL staining, Deadend Colorimetric TUNEL System (G7130, Promega) was used according to the manufacturer’s specifications. The incubation time of proteinase K was 20 min, the staining time of DAB 12 min. PAS damage scores were graded by two researchers according to Pulskens et al. (2008). Briefly, the percentage of damaged tubules in the corticomedullary junction was estimated according to the following criteria: tubular dilatation, cast deposition, brush border loss, and necrosis. Lesions were grouped into 5 groups: involvement of less than 10% of the cortex; involvement of 10%–25% of the cortex; involvement of 25%–50% of the cortex; involvement of 50%–75% of cortex; involvement of more than 75% of the cortex. TUNEL-positive nuclei were counted by two researchers. For each condition, 3* 20x microscopic images per animal were evaluated.

### 2.7 Statistics

Statistical analysis was performed with GraphPad Prism. For statistics, Shapiro-Wilk test was used to ascertain normal distribution. All data passed the Shapiro-Wilk test, except when explicitly mentioned in figure legends. Following this procedure, an unpaired two-tailed *t*-test with Welch’s correction or one-way ANOVA combined with Tukey’s multiple comparisons test were used for determination of statistical significance when data were normally distributed (see figure legends for details). If data were not normally distributed, Mann-Whitney test was used. Data were considered statistically significant if *p* < 0.05.

## 3 Results

### 3.1 Establishment of a simple, rapid and easily replicable 3D printed kidney slice incubation setup to study drug targeting of ER stress.

**FIGURE 1:**
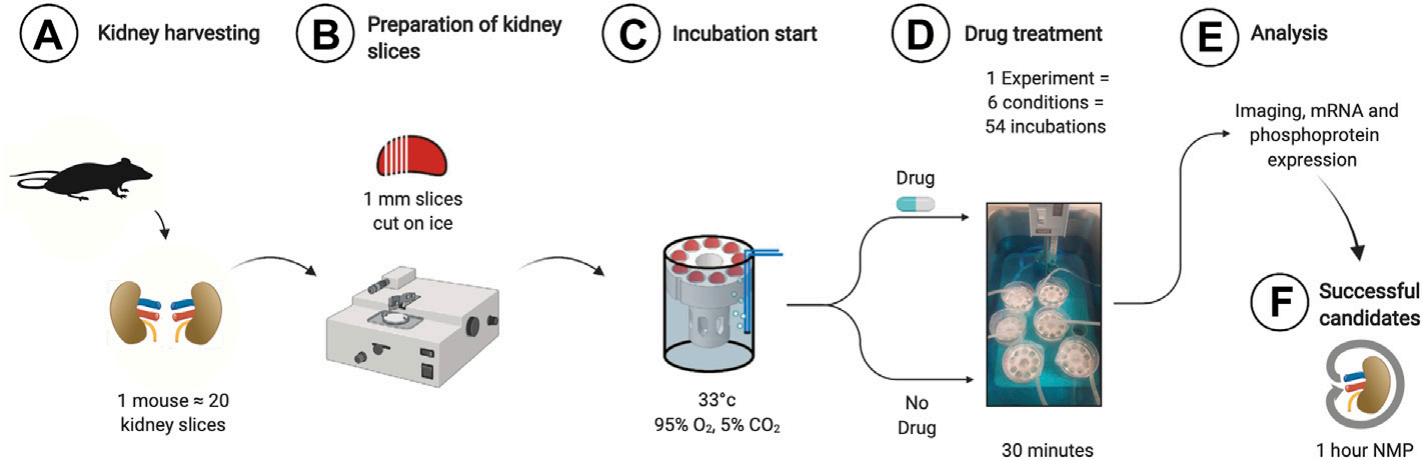
Workflow for KSI. Both kidneys are harvested and decapsulated **(A)**. On ice, ∼1 mm slices are prepared using a razor-based tissue cutter **(B)**. Slices are put into KSI incubator **(C)**. Drugs are added and incubation performed for 30 min **(D)**. Material is analyzed **(E)** and successful candidates taken towards further testing in mouse NMP.

Kidney slice incubation chambers were designed based on our previous experience (Penton et al., 2016). 3D printing allowed rapid prototyping and improvement until a final design was chosen based on the best handling properties (.stl files for reprinting in supplement). Using a razorblade-based tissue slicer, ∼20 1 mm thick kidney slices could be generated from both kidneys of one animal. The parallel use of 6 3D printed chambers allowed the investigation of *n* = 54 kidney slices incubated under 6 different conditions at the same time (Figure 1). The time spent from tissue harvesting to the end of the experiment and tissue storage was 1 hour, increasing the theoretical n/day from 2 during mouse machine perfusion to 500. First, we analyzed the optimal conditions to study ER stress during KSI. As a readout, we focused on the phosphorylation of eIF2α, since the targets of phosphorylated eIF2α appeared during the sequencing of human kidneys after NMP (Hameed et al., 2019). Analysis of eIF2α phosphorylation, a hallmark of ER stress and integrated stress response, over time showed increased phosphorylation until 30 min, which then slowly faded over time, presumably due to cell death (Using the methods described here, tissue degradation cannot be ruled out). This interpretation was underlined by conjoined loss of α-tubulin, while loading of 50 µg of protein was ensured (Supplementary Figure S1A). Using the same read-out, we tested different temperatures for incubation and arrived at 33°C.

As the optimum (Supplementary Figure S1B). Buffer and carbogen were needed to activate full eIF2α phosphorylation (Supplementary Figure S1C).

### 3.2 Kidney slice incubator and cell-free mouse kidney normothermic machine perfusion show comparable activation of the unfolded protein response

**FIGURE 2:**
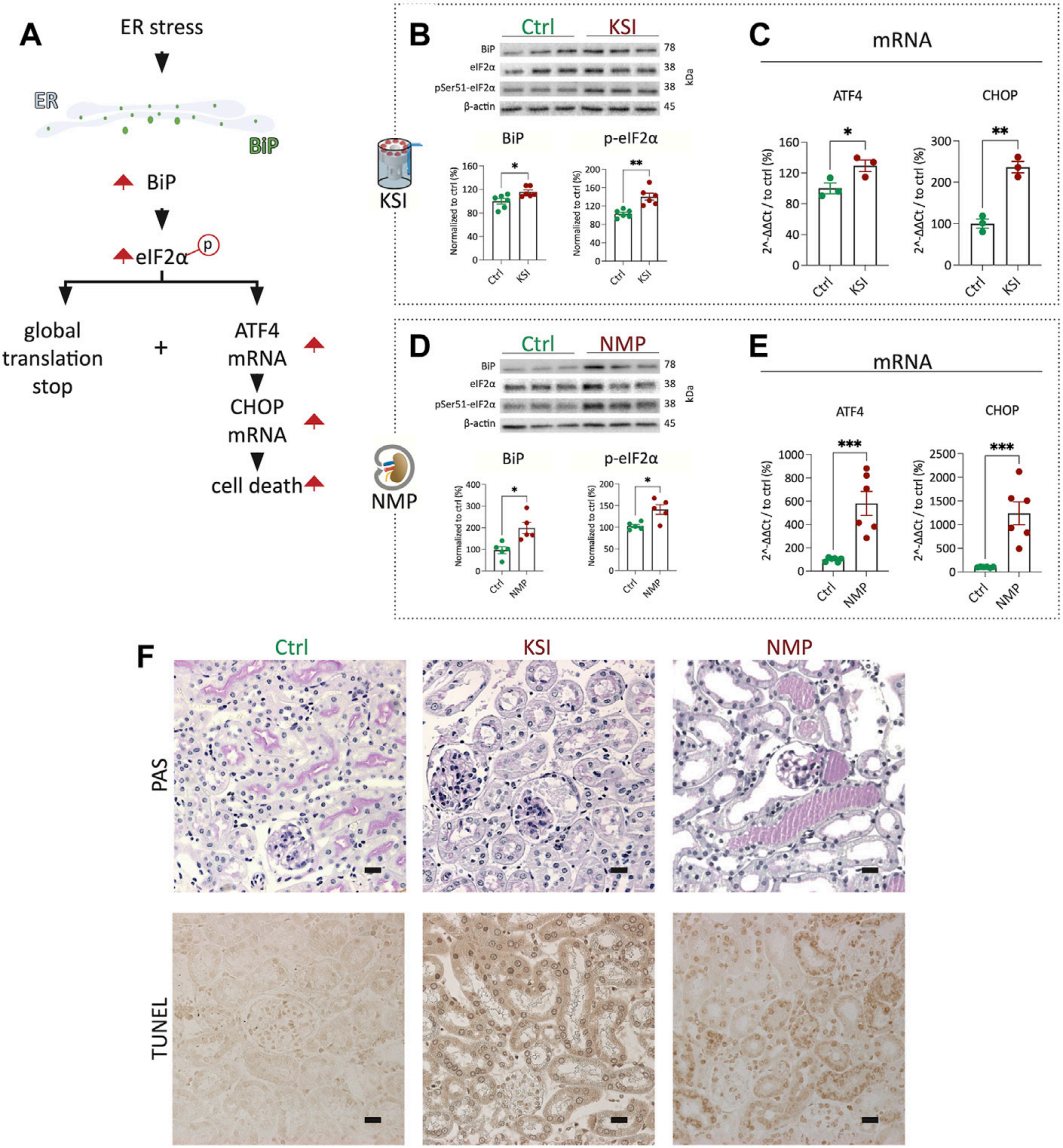
KSI and NMP exhibit broadly comparable molecular adaptions to ER stress. Pathway overview of the molecular processes of ER stress as investigated during KSI and NMP **(A)**. Western blotting reveals increased BiP release and eIF2α phosphorylation after KSI. BiP release after KSI did not pass Shapiro-Wilk test for normal distribution. **(B)**. Western blotting reveals increased BiP release and eIF2α phosphorylation after NMP **(D)**. ATF4 and CHOP mRNA are increased with KSI and NMP **(C,E)**. Increased, mainly proximal, tubular damage is seen to comparable extends after KSI and NMP using PAS- and TUNEL stainings **(F)**. Unpaired *t*-test with Welch’s correction was used for all data, except 2b BiP, where Mann-Whitney test was used. *p* values: *< 0.05, ** <0.01, ***< 0.001. Scalebar: 15 μm n ≥ 3 for KSI and n = 6 for NMP.

Next, we performed a direct comparison of UPR activation in the KSI model vs the NMP model established in our laboratory (Czogalla et al., 2016). BiP expression and eIF2α phosphorylation, hallmarks of the early UPR (For an overview of the UPR, see Figure 2A), were strongly increased after both inventions (Figures 2B,D). In both models, the mRNA of ATF4 and CHOP was upregulated, which is the classical transcriptomic response to increased eIF2α phosphorylation of eIF2 (Figures 2C,E) (Iurlaro and Muñoz-Pinedo, 2016) (Lau et al., 2013). Analysis of tubular morphology and damage after interventions revealed severe damage and TUNEL positive nuclei in proximal tubuli in both models (Figure 2F and Supplementary Figures S3B,C). Luminal PAS-positive casts, most likely resembling Tamm-Horsfall protein accumulation, underline this interpretation. In summary, UPR activation and subsequent proximal tubular damage and cell death are seen to a comparable extent in both the KSI- and the NMP models.

### 3.3 NADH ameliorates ER stress in kidney slice incubator and mouse kidney normothermic machine perfusion

**FIGURE 3:**
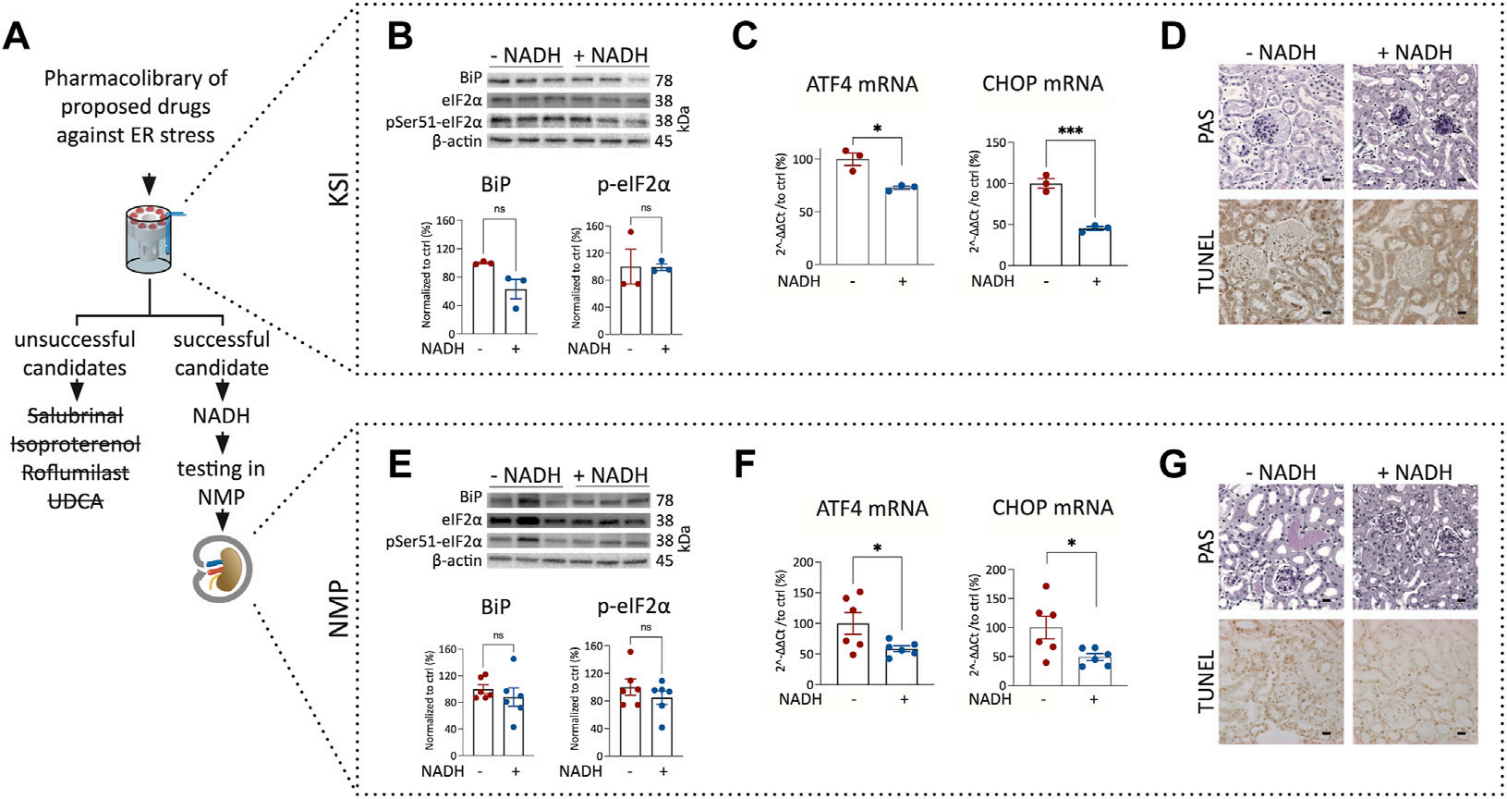
Addition of NADH reduces markers of ER stress and cell death in KSI and NMP. Workflow for drug discovery using KSI **(A)**. Western blotting reveals a nonsignificant trend towards reduced BiP release and eIF2α phosphorylation in KSI co-incubated with NADH **(B)**. ATF4 and CHOP mRNA are reduced when NADH is added to the incubation buffer **(C)** Cellular damage was less pronounced in TUNEL, but not in PAS stainings of tissue co-incubated with NADH **(D)**. BiP release and eIF2α phosphorylation in NMP co-incubated with NADH are not significantly reduced **(E)**. ATF4 and CHOP mRNA are reduced when NADH is added to the perfusion buffer **(F)** Cellular damage appears less pronounced in PAS- and TUNEL stainings of tissue coperfused with NADH **(G)**. Unpaired *t*-test with Welch’s correction was used. *p* values: *< 0.05, ** <0.01, ***< 0.001. Scalebar: 15 μm *n* = 3 for KSI and *n* = 6 for NMP.

To test whether pharmacological interventions discovered during KSI can be transferred to NMP, we investigated drugs proposed to work against ER stress (Gao et al., 2013; Chen et al., 2021; Xu et al., 2021): Salubrinal, Isoproterenol, Roflumilast, UDCA and β-Nicotinamide-adenine-dinucleotide. Surprisingly, salubrinal, isoproterenol, roflumilast, and UDCA did not show significant improvements in KSI (Figure 3A) (Supplementary Table S1). β-Nicotinamide-adenine-dinucleotide, however, did show a promising decrease in the expression of ATF4 and CHOP mRNA in KSI (Figure 3C). Initiation of apoptosis was reduced in TUNEL staining of KSI (Figure 3D and Supplementary Figure S3B). We interpreted these results as promising enough to test the addition of NADH to the perfusion buffer during NMP. When NADH was added, the effects observed during KSI were found to be reproducible (Figures 3F,G and Supplementary Figure S3C). In both models, protein analysis (Figures 3B,E) showed a trend towards reduction, however, this effect was not significant. The perfusion flow was not significantly different (Supplementary Figure S3A). To test translatability of negative results during KSI towards NMP, we tested isoproterenol addition during NMP. The addition of isoproterenol did not improve markers of ER stress during NMP (Supplementary Table S1).

## 4 Discussion

We here describe a simple, high-throughput model for drug prescreening before the time-consuming testing during mouse kidney NMP. The model can easily be reproduced using a 3D printer, allows incubation of 54 kidney slices under six different conditions at the same time, and partially recapitulates tubular damage and ER stress seen during NMP. Application of an established pharmacological treatment, β-Nicotinamide-adenine-dinucleotide, reduced transcriptomic markers of ER stress during KSI. This finding was reproducible during NMP. Isoproterenol did not reduce ER stress during KSI. This negative result was replicated during NMP. Three other unsuccessful drug candidates could be excluded before the time-consuming test during NMP.

There are several arguments against the use of mouse kidney NMP for large-scale drug testing: The method requires an advanced setup with two different pumping systems, perfusion parameters constantly need to be analyzed and adjusted, and the surgery has to be performed by specifically trained investigators. Finally, one setup allows the perfusion of only two kidneys per day, including preparation- and postprocessing time. The novel KSI model, on the other hand, has a simple setup with material found in almost any laboratory. The chambers are 3D printed and can easily be reproduced. Explantation of the kidneys does not require advanced skills. Furthermore, kidney slices, as compared to cell culture, organ-on-a-chip approaches, and kidney organoids possess the advantage of the complete renal cellular architecture (Yin et al., 2020). All in all, the KSI model provides a simple and rapid means for hypothesis-testing of drug efficacy towards ER stress before their application in NMP.

Using KSI, we found one drug, β-Nicotinamide-adenine-dinucleotide, that partially reversed the maladaptations occurring during overshooting ER stress. Given these promising results, we continued to test NADH in NMP. Underlining the value of KSI, the favorable drug response was replicable during NMP. We chose to use the NADH energy-rich NAD formulation, as we hypothesized beneficial effects during acute models. To our knowledge, this is the first time the beneficial effects of the addition of NADH to NMP preparations have been shown.

Since the molecular maladaptations we observed, as well as the morphological changes, are in line with findings in acute kidney injury (AKI), and the molecular improvement with addition of NAD has also been described for AKI *in vivo* (Arykbaeva et al., 2021) (Faivre et al., 2021; Morevati et al., 2021) (Morevati et al., 2021), KSI could additionally be a model well suited to study drug treatment for the acute phases of ischemic AKI. Other *ex-vivo* techniques for the study of ischemia-reperfusion (e.g., cell culture microflow chamber) take comparable approaches (Giraud et al., 2020). Compared to these, KSI has the advantage of intact anatomy and the disadvantage of a shorter time of observation.

There are several limitations to the KSI model and this study. We found the optimal time period for analysis of the ER stress response in KSI was 30 min. This is a shorter time than used in rodent NMP models (1 hour in mice, 2 hours in rats) (Czogalla et al., 2021). Most probably, adaptions and molecular processes occurring during NMP are missed during this shorter time. Furthermore, we found the optimal temperature for the analysis of the ER stress response in KSI is 33°C, whereas mouse kidney NMP is performed at 37°C. Although the enzyme kinetics analyzed during this study of the UPR did show roughly comparable activity, the effects seemed to be more sustained with NMP. While we focused on the analysis of one single pathway of particular interest for our laboratory, we cannot rule out that other pathways might be affected as well, however, our findings cannot be extrapolated toward other signaling pathways. During KSI, drugs reach cells via passive diffusion rather than active perfusion. While this provides a minimalistic model that can be used to study molecular signaling on the cellular level without the influence of perfusion, the perfusion itself could have various effects that are not featured in the KSI model. The number of animals chosen during KSI in this study (*n* = 3) is small and does not completely rule out that results may differ when large groups of animals are used. Lastly, we did not perform testing of all drugs under both KSI- and NMP and can thus not exclude that drugs not working in KSI would have worked in NMP.

To conclude, we developed a kidney slice model that can be used to study drug targeting of ER stress signaling and associated cell death responses. Comparison to NMP suggests that successful results may, at least in part, be transferrable. KSI provides a solution for rapid large-scale drug testing and could lead the way to generating a library of promising drug candidates for clinical testing during NMP. Further testing of NADH for clinical perfusion buffers might be warranted.

## Data Availability

The original contributions presented in the study are included in the article/Supplementary Material, further inquiries can be directed to the corresponding authors.
