# Supplementary figures and images for: A high-throughput drug discovery pipeline to optimize kidney normothermic machine perfusion
Source: Front Physiol. 2022 Sep 26;13:974615. doi: 10.3389/fphys.2022.974615 (PMC9549958; doi:10.3389/fphys.2022.974615)

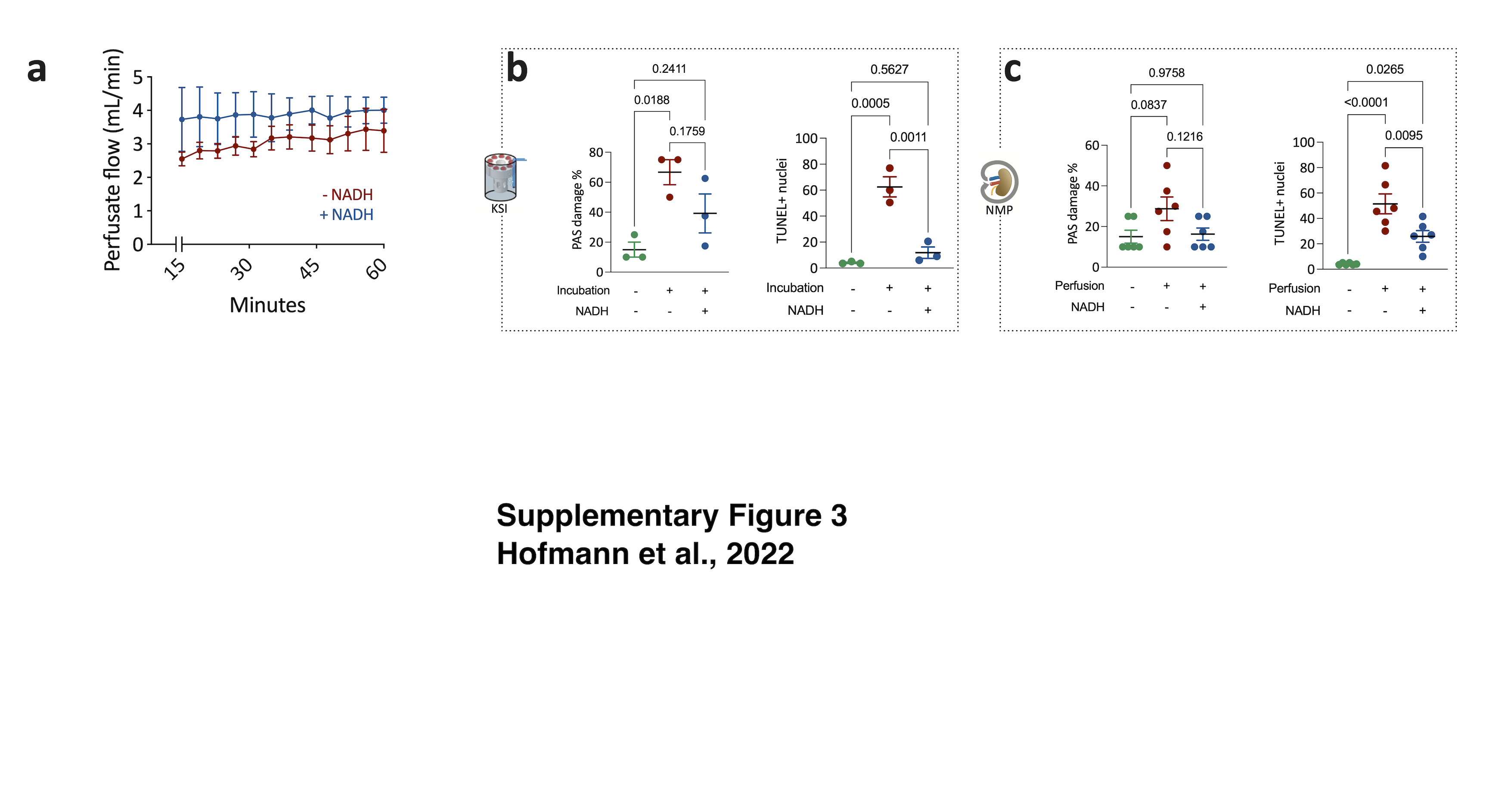

Supplement: Supplementary file 1 [file Image3.TIFF]

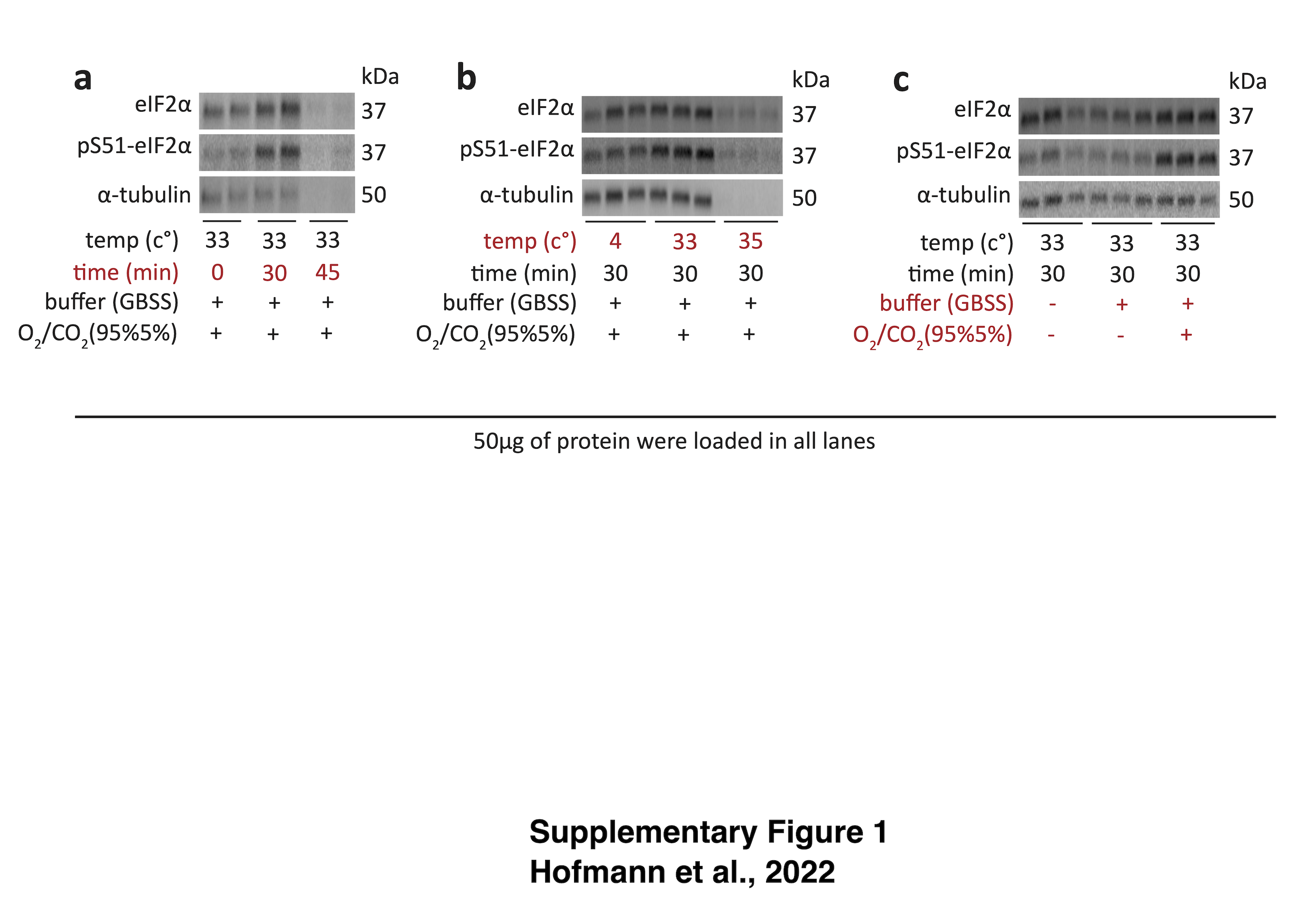

Supplement: Supplementary file 2 [file Image1.TIFF]

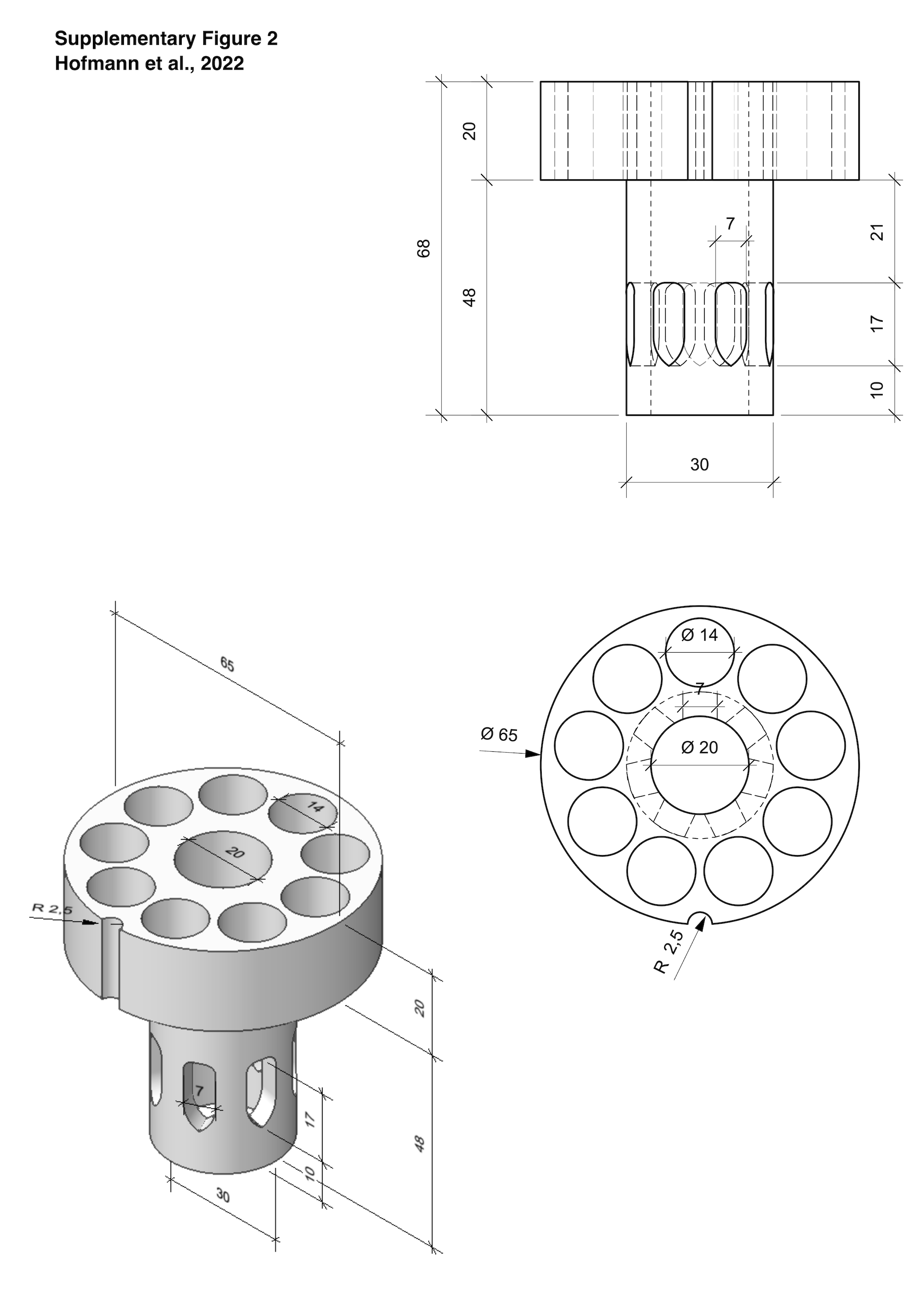

Supplement: Supplementary file 4 [file Image2.TIFF]
